# Exome and Sputum Microbiota as Predictive Markers of Frequent Exacerbations in Chronic Obstructive Pulmonary Disease

**DOI:** 10.3390/biom12101481

**Published:** 2022-10-14

**Authors:** Linfan Su, Yixian Qiao, Jinmei Luo, Rong Huang, Yi Xiao

**Affiliations:** Department of Respiratory and Critical Care Medicine, Peking Union Medical College Hospital, Chinese Academy of Medical Sciences & Peking Union Medical College, Beijing 100730, China

**Keywords:** genomics, whole-exome sequencing, chronic obstructive pulmonary disease, frequent exacerbations, 16S rRNA gene sequencing, sputum microbiome

## Abstract

Frequent acute exacerbations are the leading cause of high rates of hospitalization and mortality in chronic obstructive pulmonary disease (COPD). Despite the enormous worldwide medical burden, reliable molecular markers for effective early diagnosis and prognosis of acute exacerbations are still lacking. Both the host genetics and airway microbiome are known to play potential roles in the pathogenesis of frequent exacerbations. Here, we performed whole exome sequencing (WES) and 16S rRNA gene sequencing to explore the interaction between these two factors and their implications in the pathogenesis of frequent exacerbations. We collected peripheral blood (n = 82), sputum samples (n = 59) and clinical data from 50 frequent-exacerbation phenotype (FE) COPD patients and 32 infrequent-exacerbation phenotype (IE) as controls. Based on filtering the deleterious sites, candidate mutated genes shared only in FE patients and did not occur in the IE group were identified. Microbiota analysis revealed significant differences in bacterial diversity and composition between FE and IE groups. We report the underlying pathogenic gene including, *AATF*, *HTT, CEP350, ADAMTS9, TLL2* genes, etc., and explore their possible genotypic-phenotypic correlations with microbiota dysbiosis. Importantly, we observed that *AATF* gene mutations were significantly negatively correlated with microbial richness and diversity. Our study indicated several deleterious mutations in candidate genes that might be associated with microbial dysbiosis and the increased risk of frequent acute exacerbations in COPD patients. These results provide novel evidence that exomes and related microbiomes may potentially serve as biomarkers for predicting frequent acute exacerbations in COPD patients.

## 1. Introduction

Chronic Obstructive Pulmonary Disease (COPD) ranks among the top three causes of mortality throughout the world and contributes to increased health and economic burdens [1,2]. Acute exacerbations of COPD (AECOPD), defined as an acute worsening of respiratory symptoms that result in additional therapy, are a major cause of hospitalization and death in COPD patients [3]. As an important outcome measure, the prevention of acute exacerbations is a key target for intervention. However, there are currently no available biomarkers. So far, the best predictor is based on the patient’s recall of previous exacerbations history [4]. The frequency of exacerbations varies greatly between patients. At present, AECOPD still depends on clinical diagnosis; therefore, there is a requirement for improved diagnostic accuracy and efficiency. In particular, those COPD patients who are subject to more frequent exacerbations (two or more exacerbations per year) defined as a frequent-exacerbation phenotype, have a distinct susceptibility with worse quality of life and survival [5]. The exact reason for increased susceptibility to exacerbations remains unclear and needs to be further investigated.

Studies have shown that both genetic and environmental factors are involved in COPD pathogenesis [6,7]. In addition to alpha-1 antitrypsin deficiency (AATD), which is caused by variants in *SERPINA1* (serpin family A, member 1), there are several other genetic loci related to lung function decline or COPD risk that have been reported [8]. From candidate gene association studies to genome-wide association studies (GWASs), several genetic variants were found to be associated with the risk of COPD exacerbations [9,10]. However, research on susceptibility gene mutations for the frequent-exacerbation phenotype in COPD is limited; hence, the underlying mechanisms remain uncertain. Whole-exome sequencing (WES) was performed to identify both rare and common variants in the protein-coding portion of the human genome (less than 1% of the total genome), which has diagnostic superiority in complex polygenic disease compared with single-gene tests or whole-genome sequencing [11]. Recently, several studies have been successful in identifying functional variants for COPD risk using whole-exome sequencing [12,13]. To date, there is no relevant research investigating the frequent exacerbations involved in COPD patients.

Bacterial infections are recognized as the main triggering factor for acute COPD exacerbations [14]. However, traditional culture techniques have significant limitations and poor sensitivity [15]. With technical advances in bacterial identification, high-throughput sequencing technology such as 16S ribosomal RNA (16S rRNA) has been widely used to analyze the composition and diversity of bacterial communities in COPD [16]. A recent study using 16S rRNA gene sequencing identified that a specific sputum microbiome profile of AECOPD patients is associated with 1-year mortality and can be used to predict prognosis [17]. Despite these findings, relevant research on the frequent exacerbation phenotype and the genetics-microbiome association yet still lacking.

In this study, we selected the frequent-exacerbation phenotype in COPD as the study population. We hypothesized that rare variants or microbial dysbiosis could make the individual more susceptible to acute exacerbations. Identification of these biomarkers could provide insight into early diagnosis. To further explore the pathogenesis of this susceptibility, we performed whole-exome sequencing (WES) of the host genome and 16S rRNA gene sequencing of sputum samples to identify rare variants and microbiota differences in frequent-exacerbation phenotype COPD patients. Our findings offered an important reference for genetic basis and microbial mechanisms in COPD exacerbation susceptibility. Moreover, these findings will provide a new perspective for future diagnosis and treatment.

## 2. Materials and Methods

### 2.1. Study Design and Patient Recruitment

**Figure 1:**
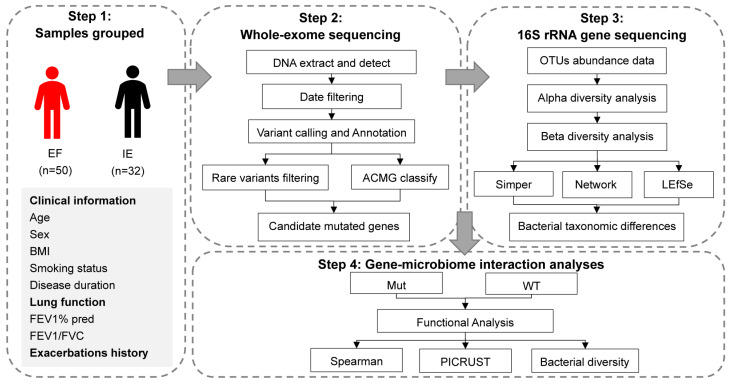
The workflow for this study.

From September 2019 to March 2021, we recruited 82 biologically unrelated Chinese COPD patients with similar ethnic backgrounds who were admitted to the Respiratory Department of Peking Union Medical College Hospital and its affiliated hospitals. A flow chart of the experimental design is shown in Figure 1. In this prospective, observational and multicentric study, inclusion criteria were as follows: (1) diagnosed with COPD according to the GOLD criteria [18]; (2) age ≥ 40 years; (3) without alpha-1 antitrypsin deficiency and a history of any other respiratory illnesses. Based on this, all the patients were classified by the frequency of exacerbations according to the past three years as frequent-exacerbation phenotype (FE, ≥2 moderate or severe exacerbations or ≥1 hospitalization for COPD exacerbation, n = 50) and infrequent-exacerbation phenotype (IE, <2 moderate exacerbations and no severe exacerbations per year, n = 32). This study was approved by the ethics committee of Peking Union Medical College Hospital (No. B306). All participants signed an informed written consent agreement.

### 2.2. Sample Collection

Peripheral blood and fresh sputum samples were collected from FE patients when admitted to the hospital for acute exacerbations and IE patients at routine outpatient visits. At the time of enrollment, peripheral blood (2 mL venipuncture) was collected from all study participants in EDTA tubes. Either spontaneous (95%) or induced (5%) sputa were collected from patients who had not received any antibiotics, oral corticosteroids, or immunosuppressive drugs 3 months before the study. Before sampling, all participants were asked to rinse their mouths with a 3% hypertonic saline solution. Induced sputum samples were then collected via inhalation of hypertonic saline (3%) for 5 to 10 min and expectoration of sputum into a sputum cup, as previously described [19]. A total of 82 peripheral blood specimens (FE, n = 50; IE, n = 32) and 59 sputum specimens (FE, n = 37; IE, n = 22) were collected and then immediately stored at −80 °C until use. The collected sputum specimens were included in the study only if they met the criteria of Gram staining (more than 25 leukocytes and fewer than 10 squamous cells per low-power microscope field). Then 0.5 mL aliquots were extracted from these valid sputum samples and stored at −80 °C for subsequent DNA extraction while the remaining part was submitted for routine bacterial culture.

### 2.3. Whole-Exome Sequencing

Genomic DNA was extracted from peripheral blood for each participant and fragmented to an average size of 180~280bp, then subjected to DNA library creation by established Illumina paired-end protocols. Agilent SureSelect Human All ExonV6 Kit (Agilent Technologies, Santa Clara, CA, USA) was used for exome capture according to the manufacturer’s instructions. To generate 150-bp paired-end reads with a minimum coverage of 10× for ~99% of the genome (mean coverage of 100×), we used the Illumina Novaseq 6000 platform (Illumina Inc., San Diego, CA, USA) for genomic DNA sequencing in Novogene Bioinformatics Technology Co., Ltd. (Beijing, China). Details are described in Scheme S1.

### 2.4. 16S rRNA Gene Sequencing

Total genome DNA from sputum samples was extracted using the CTAB method [20]. DNA concentration and purity were monitored on 1% agarose gel. DNA was diluted to 1 ng/µL using sterile water. 16S rRNA genes of distinct regions (16S V3–V4) were amplified by a specific primer in the barcode. All PCR reactions were carried out with 15 µL of the Phusion^®^ High-Fidelity PCR Master Mix (New England Biolabs, Ipswich, MA, USA); 2 µM of forward and reverse primers, and about 10 ng template DNA. Thermal cycling (initial 1 min at 98 °C 10 s) at 98 °C for 30 cycles of denaturation, annealing 30 s at 50 °C, and elongation 30 s at 72 °C; finally 72 °C for 5 min. Mix the same volume of 1× loading buffer (contained SYB green) with PCR products and operate electrophoresis on 2% agarose gel for detection. PCR products were mixed in EU density ratios. Then, the mixture of PCR products was purified with Qiagen Gel Extraction Kit (Qiagen, Hilden, Germany). Sequencing libraries were generated using TruSeq^®^ DNA PCR-Free Sample Preparation Kit (Illumina, San Diego, CA, USA) following the manufacturer’s recommendations and index codes were added. The library quality was assessed on the Qubit@ 2.0 Fluorometer (Thermo Scientific, Waltham, MA, USA) and Agilent Bioanalyzer 2100 system. At last, the library was sequenced on an Illumina NovaSeq platform with 250 bp paired-end reads generated. Details are described in Scheme S1.

### 2.5. Bioinformatics Analysis

For WES, rare variants filtering was performed as follows: (1) SNPs with a minor allele frequency (MAF) less than 0.01 in 1000 genomic data (1000g_all), esp6500siv2_all (http://evs.gs.washington.edu/EVS; accessed on 8 April 2021), gnomAD data (gnomAD_ALL/gnomAD_EAS, https://doi.org/10.1101/030338; accessed on 8 April 2021) and Novo-Zhonghua exome database (an in-house Chinese-population exome database); (2) Only SNVs occurring in exons or splice sites (splicing junction 10 bp) were further analyzed; (3) Both synonymous SNVs (not relevant to the amino acid alternation predicted by dbscSNV) and small fragment non-frameshift (<10 bp) indel in the repeat region defined by RepeatMasker were discarded. (4) Variations were screened according to prediction scores of software including SIFT (v6.2.0), Poly Phen (v2.2.2), MutationTaster (v2013), and CADD (v1.3). Potentially deleterious variations are reserved for at least half of these four software supporting harmfulness of variations. For 16s rRNA gene sequencing, sequence analysis was performed by Uparse software (Uparse v7.0.1001, http://drive5.com/uparse/; accessed on 20 May 2021). Alpha diversity was applied in analyzing the complexity of species diversity indices calculated with QIIME (Version 1.7.0) and displayed with R software (Version 2.15.3). Principal Coordinate Analysis (PCoA) was displayed by the WGCNA package, stat packages, and ggplot2 package in R software (Version 2.15.3). Microbial functionality profiles were predicted using Phylogenetic Investigation of Communities by Reconstruction of Unobserved States (PICRUSt) to generate the Kyoto Encyclopedia of Genes and Genomes (KEGG) pathways [21].

### 2.6. Statistical Analysis

SPSS 21.0 software was used for basic statistical analysis of data, and GraphPad Prism9 (version 9.0.0) and R software (Version 2.15.3) were used for chart production (*p* < 0.05 was considered statistically significant). Normally distributed variables were presented as mean ± standard deviation and compared through one-way analysis of variance (ANOVA) followed by Tukey’s multiple comparison test. The nonparametric data were presented as median (interquartile range) and were analyzed by Mann-Whitney tests. Categorical variables were expressed as count and percentages (N, %) and compared using the χ^2^ test. Spearman’s correlation was used to determine the relationship between the mutated genes and the number of acute exacerbations per year.

## 3. Results

### 3.1. Clinical Characteristics

We recruited 82 patients, including 67 male and 15 female individuals. The average age at onset and inclusion are 60.44 and 69.77 years, respectively. Furthermore, there were 50 and 32 cases of frequent exacerbator and infrequent exacerbator, with no statistically significant difference in age, sex, BMI, smoking status, COPD duration, and GOLD classification between the groups (Table 1). The results of sputum culture showed no statistical difference may be due to the low positive rate. As expected, the frequent exacerbator group showed a significant difference in the exacerbations frequency (*p* < 0.001) and the resulting impaired lung function (*p* = 0.009).

### 3.2. Whole Exome Sequencing Analyses

We performed WES on 82 DNA samples using the Illumina platform. On average, the sequencing quality values of Q30 (percentage of bases with Phred value > 30) and Q20 were above 80 and 90%, respectively, with a sequencing error rate less than 0.1%, indicating that the sequencing was reliable. A total of 22,308 loci for single nucleotide variants (SNVs) and 677 loci for insertions and deletions (INDELs) were identified, including 11,366 synonymous SNV, 10,316 missense mutations, 88 stopgain, 14 stoploss, and 524 unknown; as well as, 90 frameshift deletions, 72 frameshift insertions, 208 nonframeshift deletions, 200 nonframeshift insertions, 2 stoploss, 9 stopgain, and 96 unknown.

To identify potentially pathogenic variants, we screened variations according to scores of algorithms including SIFT (v6.2.0), Polyphen (v2.2.2), Mutation Taster (v2013), and CADD (v1.3) software. Potentially deleterious variations were reserved if more than half of these software scores supported the harmfulness of variations. With 376 candidate genes identified, the top 20 mutated genes were provided as Supplementary Materials in Figure S1. Based on filtering the deleterious sites, we screened the control group (IE) for allelic mutations and determined several genes were only expressed in FE patients (Table 2) and the *AATF* gene had the highest rank. The following top five most frequent genes only mutated in the FE group: *AATF* (18%), *HTT* (16%), *CEP350* (14%), *ADAMTS9* (14%), and *TLL2* (14%). Strong correlations were found in the number of acute exacerbations per year (|*r*| > 0.3, *p* < 0.05) instead of FEV1% pred (|*r*| < 0.3, *p* = 0.803) between *AATF* gene.

### 3.3. Sputum Microbiota Profiling

We investigated sputum microbiota on 59 sputum specimens (FE, n = 37; IE, n = 22) using 16S rRNA V3–V4 amplicon sequencing. Bacterial diversity and compositions in different COPD phenotypic subgroups were first compared. Microbial diversity, according to the Shannon index and Simpson index, was lower in the FE group (*p* < 0.01), indicating that the sputum microbiota in these patients was characterized by a lower diversity than those in the IE patients. A principal coordinates analysis (PCoA) showed significant differences in sputum microbiota between the FE and IE groups (Supplementary Materials in Figure S2).

**Figure 2:**
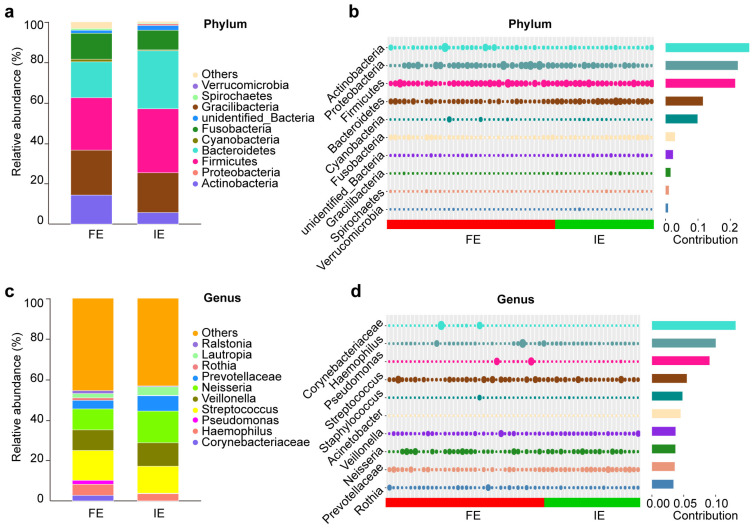
Bacterial community structure in FE (n = 37) and IE (n = 22) group. Relative abundance and Simper analysis of the most prevalent bacteria and at the phylum (**a**,**b**) and genus levels (**c**,**d**) in FE and IE group. The bubble size represents the relative abundance of the species, and the contribution represents the contribution of the species in the two groups of differences. FE frequent exacerbator, IE infrequent exacerbator.

At the phylum level, the numbers of phyla detected in the FE and IE groups were 35, and 22, respectively (Figure 2c). The dominant bacterial phylum in the FE group was Firmicutes (30.20%), followed by Proteobacteria (29.49%), and Bacteroidetes (13.69%). In the IE group, the major phylum was Firmicutes (31.61%), followed by Bacteroidetes (28.85%), and Proteobacteria (19.64%). The similarity percentage (SIMPER) analysis indicated that Actinobacteria, Proteobacteria, and Firmicutes explained most of the differences in community structure among FE and IE groups (Figure 2d).

At the genus level, we detected 566 and 363 genera in the FE and IE groups, respectively (Figure 2). The dominant genera in the FE group were *Streptococcus* (15.63%), *Neisseria* (12.02%), and *unidentified_Prevotellaceae* (8.48%). In the IE group, the dominant genera were *unidentified_Prevotellaceae* (15.62%), *Streptococcus* (13.30%), and *Neisseria* (11.65%). SIMPER analysis indicated that the major dissimilarity contributors were *Corynebacteriaceae*, *Haemophilus,* and *Pseudomonas* (Figure 2).

### 3.4. Microbiome Network

**Figure 3:**
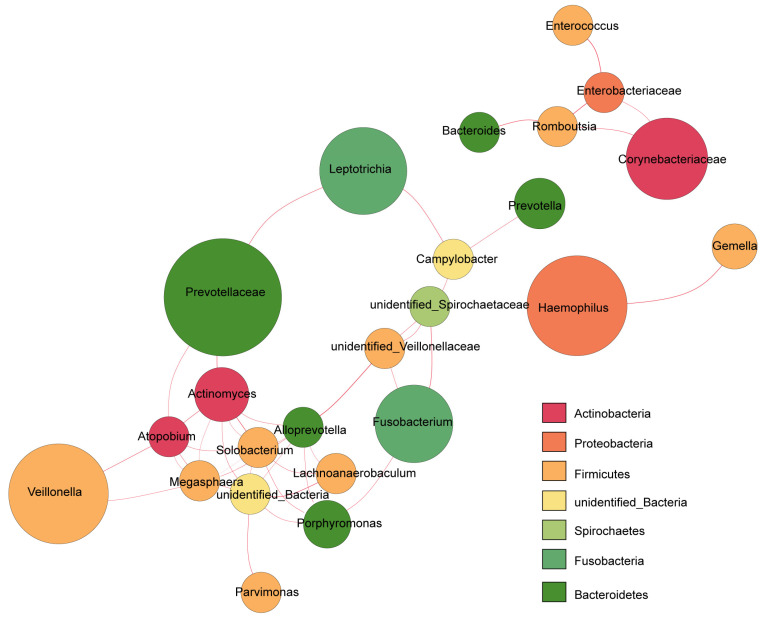
Network of Bacteria in FE group by Spearman rank correlation analysis. Different nodes represent different genera, the node size represents the degree of connectivity of the genus, and the same color represents the level of the same gate (as shown in the legend). The thickness of the connecting line between nodes is positively correlated with the absolute value of the correlation coefficient of species interaction. Regarding the color of the line, red represents a positive correlation.

To explore the potential bacterial co-existence relationships in the FE Mut group, we performed an interaction network analysis. We selected the most abundant phyla and the specific network was built and estimated based on the relative abundances using Spearman correlation analysis (Figure 3). In the FE group, *Ralstonia* was included in a closed positively correlated network containing *Veillonella, Prevotellaceae, Porphyromonas,* and *Fusobacterium*. In addition, *Haemophilus* showed a positive correlation with *Gemella*.

### 3.5. Correlation between Candidate Genes and the Microbiome

**Figure 4:**
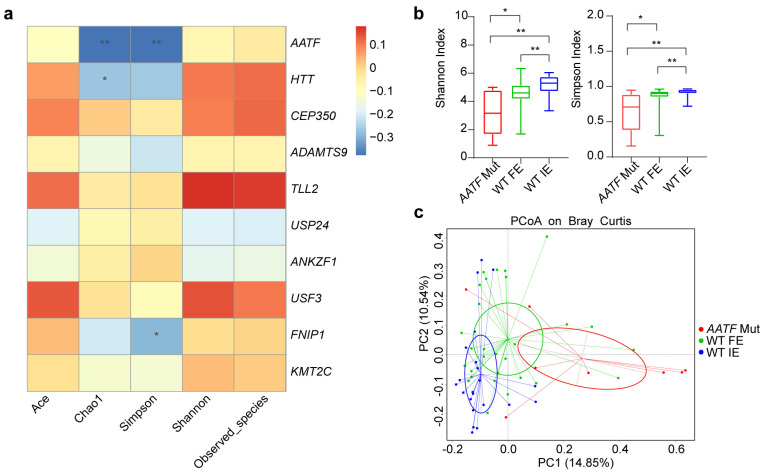
(**a**) Heatmap of Spearman correlation analysis between the relative abundances of sputum microbiome and alpha diversity indices. Significant association (|*r*| > 0.3, *p* < 0.01) were shown between Chao1 index and Simpson index in *AATF* gene. (**b**) Comparison of alpha diversity indices (Shannon index, Simpson’s index) of *AATF* Mut (n = 9), WT FE (n = 28), and WT IE (n = 22) by Wilcoxon rank-sum test. (**c**) PCoA is based on Bray-Curtis’s dissimilarity. ADONIS analysis showed that the separation of bacterial communities was significant (*AATF* Mut vs WT IE, *p* = 0.001; *AATF* Mut vs WT FE, *p* = 0.001; WT FE vs WT IE, *p* = 0.001;). * *p* < 0.05, ** *p* < 0.01. Mut, mutation; WT, wild-type.

Spearman’s correlation analysis was used to evaluate the correlation between alpha diversity and major mutated genes only detected in the FE group. As shown in Figure 4a, we found a significant association (|*r*| > 0.3, *p* < 0.01) between *AATF* gene mutation and decreased microbial richness (Chao1 index) and microbial diversity (Simpson index).

We further sub-grouped subjects into FE patients with *AATF* Mutations (*AATF* Mut), AATF wild-type in FE patients (WT FE), and AATF wild-type in IE patients (WT IE). *AATF* Mut group had the lowest alpha-diversity indices compared to the other groups and beta-diversity by PCoA analysis showed that groups had significantly different overall taxonomic compositions (Figure 4b,c).

### 3.6. Bacterial Taxonomic Differences

**Figure 5:**
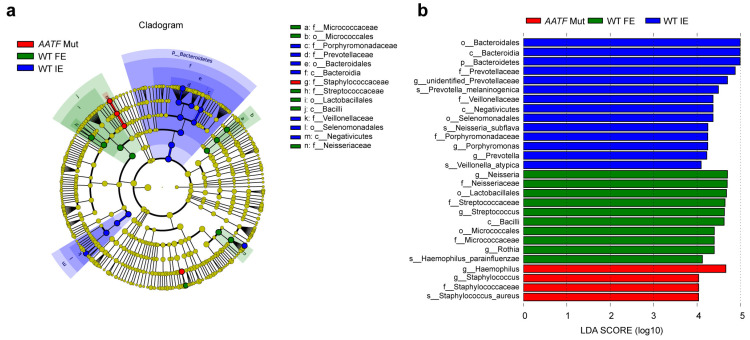
Linear discriminant analysis effect size (LEfSe) analysis revealed the discriminative microbes. LEfSe cladogram (**a**) and histogram of linear discriminant analysis (LDA) score (**b**) show the biomarkers with significant differences groups, with LDA score > 4.0. The circle radiating from inside to outside represents the classification from the phylum to the genus. Each circle’s diameter represents the relative abundance and different species biomarker follows the group for coloring.

To further identify bacterial biomarkers between groups, we performed a LEfSe analysis at the genera level (Figure 5a,b). The discriminative bacteria identified in the *AATF* Mut group were *Haemophilus* and *Staphylococcus*; whereas, the microbiome in the WT FE and WT IE group was dominated by the genus *Neisseria*, *Streptococcus* and *Porphyromonas*, *Prevotella, Veillonella*, etc. (*p* < 0.05).

### 3.7. Functional Analysis of the Microbiome in FE-Associated Genes by PICRUSt Analysis

**Figure 6:**
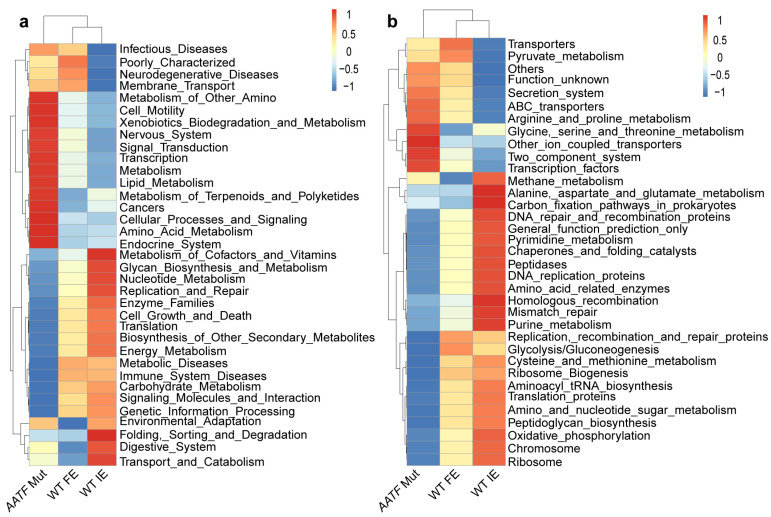
The differences in metabolic pathways by PICRUSt functional analysis. Related KEGG pathways are plotted at KEGG level 2 (**a**), and KEGG level 3 (**b**) between three groups. Mut, mutation; WT, wild-type.

Additionally, we conducted a Phylogenetic Investigation of Communities by Reconstruction of Unobserved States (PICRUSt) analysis based on the Kyoto Encyclopedia of Genes and Genomes (KEGG) database to predict the microbiota’s metabolic functions across groups in our study cohort. Specifically, we found that the levels of genetic information processing and translation, such as replication and repair, membrane transport, and ABC transporters, were more enriched in the *AATF* Mut group than in the *AATF* WT group. In contrast, metabolism related to biosynthesis and degradation including glycan, amino acid, and xenobiotics, were decreased in the *AATF* Mut group (*p* < 0.05; Figure 6a,b).

## 4. Discussion

COPD is a complex, heterogeneous polygenic disease that is influenced by both heredity and the environment. Frequent-exacerbation COPD, one of the most important phenotypes, has been reported with genetic susceptibility and lung microbial characteristics; however, the underlying mechanisms were yet to be investigated. In the present study, we hypothesized that these frequent-exacerbation phenotypes may carry acute exacerbations susceptibility gene mutations and microbial dysbiosis comparable to stable COPD phenotype. Using whole-exome sequencing and 16S rRNA gene sequencing, we first identified the most frequently mutated genes and specific microbial communities that were highly likely to increase exacerbation risk. We also explored the potential association between mutated genes and microbial differences by functional analysis. Together, the current study sheds some new light on the potential of certain exosomes and sputum microbes as diagnostic biomarkers or therapeutic targets for frequent exacerbators in COPD patients.

Our results demonstrated reduced microbial diversity in the FE group, compared with the IE group. Similar trends were also observed in COPD exacerbations compared to stable states, which is consistent with our findings [22]. Reduced sputum bacterial diversity has been reported to be associated with more severe airflow obstruction and associated with an outgrowth of potentially pathogenic bacteria [23,24]. During exacerbations, pathogenic bacteria may hold dominant positions relative to other species and accelerate declines in microbial diversity. Previous studies have shown that reduced bacterial diversity evaluated with 16S rRNA gene sequencing is associated with disease severity and 1-year mortality [17,25]. So far, despite no specific cut-off values, sputum bacterial diversity could be used for evaluating disease progression and prognosis, reflecting the dynamic changes during exacerbations in COPD patients. We also observed significant differences in the microflora structure between FE and IE patients. At the phylum level, the most dominant phyla in sputum samples of both IE and FE groups was Firmicutes. The main difference is that Actinobacteria and Proteobacteria accounted for more presentations in the FE group than in the IE group. Firmicutes and Actinobacteria were commonly found in the sputum of COPD patients [26]. Proteobacteria has been confirmed to be associated with exacerbation events [27]. At the genus level, *Streptococcus* was the most predominant genera at FE, whereas *Prevotellaceae* was dominant at IE. *Corynebacteriaceae* and *Haemophilus* contribute the most to variance in the FE group. LEfSe analysis discriminative bacteria in the FE group was *Haemophilus*, whereas the microbiome in the IE COPD group was dominated by the genus *Porphyromonas, Prevotella,* and *Veillonella*. Previous studies have shown that *Haemophilus influenzae* and *Streptococcus pneumoniae* are the most common pathogenic bacterium involved in acute exacerbations of COPD [28]. Although they can be identified in stable states, their relative loads were considerably higher during exacerbations [29]. The bacterial co-existence interaction network maps showed that relatively stable lung bacterial flora is considered part of the “core pulmonary microbiome” [30]. When the balance of pulmonary microbial flora is broken, the biological barrier is disrupted and exacerbations occur. In general, our study further suggests changes in bacterial diversity and abundance may lead to acute exacerbations and can be used as future biomarkers.

Among the candidate genes in the present study, the apoptosis-antagonizing transcription factor (*AATF*) gene was identified as the most susceptibility gene associated with a predisposition to frequent exacerbation risk. Acting as a key molecule to sustain proliferative tissues and tumor progression in parts, *AATF* was reported as a transcriptional regulator in inhibiting p53-driven apoptosis in vivo [31]. *AATF* has also been reported overexpressed in various cancerous tissues, including lung and colorectal cancer, as well as lymphomas [32]. A recent study revealed *AATF* as one of the candidate genes associated with acute lung injury [33]. It has been reported that *AATF* expression increases during disease progression and is involved in a pathway related to airway inflammation in COPD [34,35]. However, the mechanism behind this remains unknown.

Recent research has confirmed that several gene mutations affect the regulation of the gut microbiota and may participate in the pathogenesis of digestive tract diseases [36]. To the best of our knowledge, there are currently no other studies involving host genetic variation and sputum microbiome in COPD patients. In this study, we explored for the first time the sputum microbiome characteristic of COPD patients with FE-related gene mutations. Correlation analysis suggested that *AATF* mutation status was significantly negatively correlated with microbial diversity and richness. Compared to patients with wild-type *AATF*, alpha diversity was significantly decreased in the *AATF* mutant group. Reduced bacterial diversity was associated with an outgrowth of potentially pathogenic bacteria as mentioned before, which indicated that the *AATF* mutant group was more susceptible to bacterial infections. Further, functional prediction showed that bacterial genetic signal transduction was enriched while biosynthesis and degradation metabolism decreased in the *AATF* mutant group. Signal transduction pathways that regulate the host-pathogen interactions play important roles in the pathogenesis of bacterial infection [37]. Decreased metabolic pathways related to lipid metabolism, glycan biosynthesis, and metabolism and amino acid metabolism have been previously reported to be associated with microbiome shift and energy consumption [38]. All of the above results suggested a more active bacterial infection. It can be speculated that *AATF* mutant in frequent exacerbators may be more genetically susceptible to airway inflammation, and harbor more active bacterial infection that may contribute to deterioration. Further transcriptome and proteome analyses of the microbiome are needed to clarify the underlying mechanisms.

One of the major limitations of the present study is the relatively small sample size; therefore, a larger follow-up study is needed subsequently to validate our findings. Another shortcoming is that our analyses were limited to individuals of Chinese ancestry and may therefore limit the generalizability. Additionally, the WES-based method cannot detect genomic variants in non-protein-coding regions or structural variants that may affect AECOPD risk. Another potential limitation is that we could not obtain sputum samples from all patients because some participants failed to produce sputum samples or the sputum samples didnot meet the standards. We believe in our subsequent studies, these limitations will be overcome. Nevertheless, our study also has several strengths, such as the use of human peripheral blood samples, multi-omics, prospective case-control study design, and significantly higher frequencies of the *AATF* gene found in the frequent exacerbator group.

## 5. Conclusions

In conclusion, variants and microbial signatures identified in this study have important implications for the prevention and management of COPD exacerbations in frequent-exacerbation phenotype. However, these findings should be generalized with caution until validated in larger cohorts. The underlying molecular and biochemical mechanisms of genes susceptibility and microbial dysbiosis to acute exacerbation in COPD patients deserve further investigations to clarify in the future.

## Figures and Tables

**Table 1:** Demographic characteristics of the COPD patients.

Variables	All	Infrequent Exacerbator	Frequent Exacerbator	*p*-Value
n	82	32	50	
Age at inclusion (years) (mean ± SD)	69.77 ± 8.14	67.91 ± 7.97	70.96 ± 8.18	0.100
Age at onset of COPD (years)	60.44 ± 8.83	59.28 ± 6.31	61.18 ± 10.19	0.348
COPD duration, years (median, range)	10.00 (5.00–10.00)	7.50(3.50–10.00)	10.00 (5.00–11.25)	0.513
Sex, male, n (%)	67 (81.71)	28 (87.50)	39 (78.00)	0.278
BMI (kg/m^2^) (median, range)	23.29 (21.50–25.95)	24.49 (22.15–26.42)	22.88 (21.16–25.97)	0.116
Current smoker No. (%)	19 (23.17)	9 (28.13)	10 (20.00)	0.395
FEV1% pred	53.38 ± 20.54	60.74 ± 22.34	48.67 ± 18.23	**0.009**
FEV1/FVC	52.05 ± 12.42	51.10 ± 13.56	52.73 ± 11.81	0.577
Sputum culture positive, n (%)	6 (7.3)	1 (3.1)	5 (10.0)	0.244
Acute exacerbation (median, range)	1.00 (0.00–2.00)	0.00 (0.00–1.00)	2.00 (1.00–3.00)	**<0.001**
GOLD Classification, n (%)				0.508
Ⅰ	13 (15.9)	7 (21.9)	6 (12.0)	
Ⅱ	32 (39.0)	13 (40.6)	19 (38.0)	
Ⅲ	25 (30.5)	9 (28.1)	16 (32.0)	
Ⅳ	12 (14.6)	3 (9.4)	9 (18.0)	

COPD: chronic obstructive pulmonary disease; BMI: body mass index; FEV1: forced expiratory volume in 1 s; FVC: forced vital capacity; GOLD: global initiative for obstructive lung disease; SD: standard deviation. Significant differences are in bold.

**Table 2:** The top 10 mutated genes were only detected in the FE group.

CHR	Gene Name	Description	Exon Variant Types	Frequency in FE	Frequency in IE	Expression Summary	*r*	*p*-Value
17	*AATF*	Apoptosis-antagonizing transcription factor	Missense	9/50	0/32	Ubiquitous granular cytoplasmic expression	0.463	**<0.001**
4	*HTT*	Huntingtin	Missense, Nonframeshift insertion	8/50	0/32	Cytoplasmic expression in most tissues	0.246	**0.026**
1	*CEP350*	Centrosomal protein 350	Missense, Nonframeshift deletion	7/50	0/32	Ubiquitous cytoplasmic expression	0.051	0.648
3	*ADAMTS9*	ADAM metallopeptidase with thrombospondin type 1 motif 9	Missense	7/50	0/32	Cytoplasmic expression in most tissues at variable levels	0.193	0.082
10	*TLL2*	Tolloid-like 2	Nonframeshift insertion	7/50	0/32	NA	0.221	**0.046**
1	*USP24*	Ubiquitin-specific protease 24	Stopgain	6/50	0/32	Cytoplasmic expression in most cell types	0.192	0.084
2	*ANKZF1*	Ankyrin repeat- and zinc finger domain-containing 1	Frameshift deletion	6/50	0/32	Cytoplasmic expression in all tissues	0.213	0.055
3	*USF3*	Upstream transcription factor family, member 3	Missense	6/50	0/32	NA	0.102	0.363
5	*FNIP1*	Folliculin-interacting protein 1	Missense	6/50	0/32	Mainly cytoplasmic expression at variable levels in all cell types	0.162	0.147
7	*KMT2C*	Lysine (K)-specific methyltransferase 2C	Missense	6/50	0/32	NA	0.205	0.065

CHR: Chromosome, Exon variant types variant type of exon region: Frequency in FE/IE: The number of mutation sites shared by frequent-exacerbation or infrequent-exacerbation groups, Expression summary: the expression and distribution of genes in different tissues and at the subcellular level, NA: not applicable. Significant differences are in bold.

## Data Availability

The data presented in this study are available on request from the corresponding author.

## Supplementary Materials

The following supporting information can be downloaded at: https://www.mdpi.com/article/10.3390/biom12101481/s1. Figure S1: Mutational profile of top 20 mutated genes in candidate genes; Figure S2: Bacterial community structure in FE (n = 37) and IE (n = 22) group; Scheme S1: Whole-exome Sequencing and 16S rRNA gene sequencing.

## Abbreviations

COPD: chronic obstructive pulmonary disease; AECOPD: Acute exacerbations of chronic obstructive pulmonary disease; WES: whole exome sequencing; GWASs: genome-wide association studies; FE: frequent exacerbation; IE: infrequent-exacerbation; SNVs: Single nucleotide variants; INDELs: insertions and deletions; BMI: body mass index; FEV1: forced expiratory volume in 1 s; FVC: forced vital capacity; GOLD: global initiative for obstructive lung disease; SD: standard deviation; NA: not applicable; PCoA: Principal coordinates analysis; LEfSe: Linear discriminant analysis effect size; LDA: Linear discriminant analysis; WT: wild-type; Mut: Mutations; KEGG: Kyoto Encyclopedia of Genes and Genomes; PICRUSt: Phylogenetic Investigation of Communities by Reconstruction of Unobserved States; AATF: apoptosis-antagonizing transcription factor.
