# Supplementary material for: Exome and Sputum Microbiota as Predictive Markers of Frequent Exacerbations in Chronic Obstructive Pulmonary Disease
Source: Biomolecules. 2022 Oct 14;12(10):1481. doi: 10.3390/biom12101481 (PMC9599557; doi:10.3390/biom12101481)
Supplement: Supplementary file 1 [file biomolecules-12-01481-s001.zip › biomolecules-1919402-Scheme S1.pdf]

Whole-exome Sequencing

1. Sequencing and data analysis

DNA extract and detect

Genomic DNA extracted from peripheral blood for each sample was fragmented to an average size of 180~280bp and subjected to DNA library creation using established Illumina paired-end protocols. The Agilent SureSelect Human All ExonV6 Kit (Agilent Technologies, Santa Clara, CA, USA) was used for exome capture according to the manufacturer's instructions. The Illumina Novaseq 6000 platform (Illumina Inc., San Diego, CA, USA) was utilized for genomic DNA sequencing in Novogene Bioinformatics Technology Co., Ltd (Beijing, China) to generate 150-bp paired-end reads with a minimum coverage of 10× for ~99% of the genome (mean coverage of 100×).

Data analysis

After sequencing, basically files conversion and demultiplexing were performed with bcl2fastq software (Illumina). The resulting fastq data were submitted to in-house quality control software for removing low quality reads, and then were aligned to the reference human genome (hs37d5) using the Burrows-Wheeler Aligner (bwa), and duplicate reads were marked using sambamba tools.

SNP/INDEL calling

Single nucleotide variants (SNVs) and indels were called with samtools to generate gVCF. The raw calls of SNVs and INDELs were further filtered with the following inclusion thresholds: 1) read depth > 4; 2) Root-Mean-Square mapping quality of covering reads > 30; 3) the variant quality score > 20.

CNV calling

The copy number variants (CNVs) were detected with software CoNIFER (V0.2.2).

Annotation

Annotation was performed using ANNOVAR (2017June8). Annotations included minor allele frequencies from public control data sets as well as deleteriousness and conservation scores enabling further filtering and assessment of the likely pathogenicity of variants.

2. Rare variants filtering

Filtering of rare variants was performed as follows: (1) variants with a MAF less than 0.01 in 1000 genomic data (1000g_all), esp6500siv2_all, gnomAD data (gnomAD_ALL and gnomAD_EAS) and in house Novo-Zhonghua exome database from Novogene; (2) Only SNVs occurring in exons or splice sites (splicing junction 10 bp) are further analyzed since we are interested in amino acid changes. (3) Then synonymous SNVs which are not relevant to the amino acid alternation predicted by dbscSNV are discarded; The small fragment non-frameshift (<10bp) indel in the repeat region defined by RepeatMasker are discarded. (4) Variations are screened according

to scores of SIFT, Polyphen, MutationTaster and CADD softwares. The potentially deleterious variations are reserved if the score of more than half of these four softwares support harmfulness of variations. Sites(>2bp) did not affect alternative splicing were removed.

3. ACMG classify

In order to better predict the harmfulness of variation, the classification system of the American College of Medical Genetics and Genomics (ACMG) was used. The variations are classified into pathogenic, likely pathogenic, uncertain significance, likely benign and benign.

16S rRNA gene sequencing

Data analysis

1. Paired-end reads assembly and quality c ontrol

1.1 Data split

Paired-end reads was assigned to samples based on their unique barcode and truncated by cutting off the barcode and primer sequence.

1.2 Sequence assembly

Paired-end reads were merged using FLASH (V1.2.7, http: //ccb.jhu.edu/software/FLASH/), a very fast and accurate analysis tool, which was designed to merge paired-end reads when at least some of the reads overlap the read generated from the opposite end of the same DNA fragment, and the splicing sequences were called raw tags.

1.3 Data Filtration

Quality filtering on the raw tags were performed under specific filtering conditions to obtain the high-quality clean tags according to the QIIME(V1.9.1, http://qiime.org/scripts/split_libraries_fastq.html) quality controlled process.

1.4 Chimera removal

The tags were compared with the reference database(Silva database,using UCHIME algorithm(UCHIME http://www.drive5.com/usearch/manual/uchime_algo.html) to detect chimera sequences, and then the chimera sequences were removed. Then the Effective Tags finally obtained.

2. OTU cluster and Species annotation

2.1 OTU Production

Sequences analysis were performed by Uparse software (Uparse v7.0.1001, http://drive5.com/uparse/). Sequences with ≥ 97% similarity were assigned to the same OTUs.Representative sequence for each OTU was screened for further annotation.

2.2 Species annotation

For each representative sequence, the Silva Database (http://www.arb-silva.de/)was used based on Mothur algorithm to annotate taxonomic information.

2.3 Phylogenetic relationship Construction

In order to study phylogenetic relationship of different OTUs, and the difference of the dominant species in different samples (groups), multiple sequence alignment were conducted using the MUSCLE software (Version 3.8.31,

http://www.drive5.com/muscle/).

2.4 Data Normalization

OTUs abundance information were normalized using a standard of sequence number corresponding to the sample with the least sequences. Subsequent analysis of alpha diversity and beta diversity were all performed basing on this output normalized data.
