# Supplementary material for: Exome and Sputum Microbiota as Predictive Markers of Frequent Exacerbations in Chronic Obstructive Pulmonary Disease
Source: Biomolecules. 2022 Oct 14;12(10):1481. doi: 10.3390/biom12101481 (PMC9599557; doi:10.3390/biom12101481)
Supplement: Supplementary file 1 [file biomolecules-12-01481-s001.zip › biomolecules-1919402-supplementary.pdf]

biomolecules

MDPI

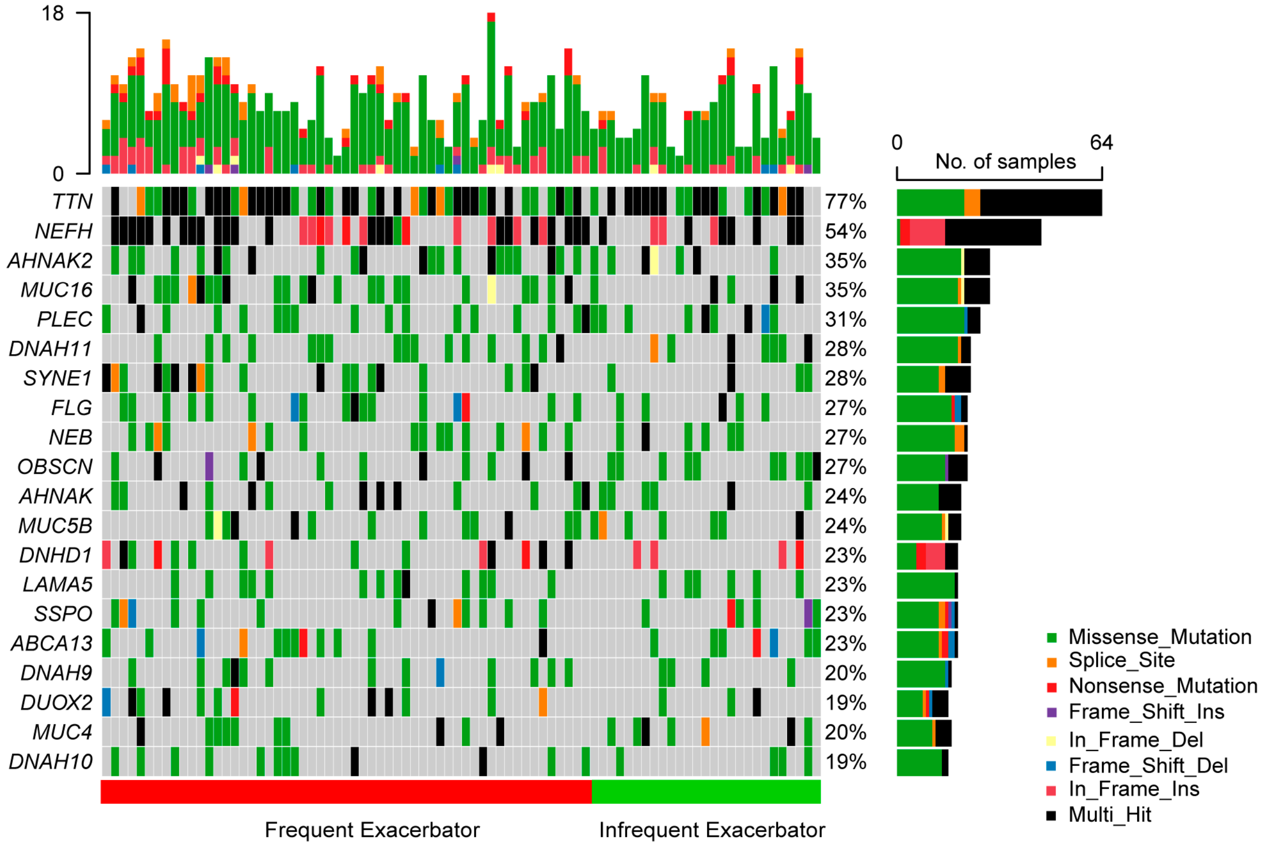

Figure S1. Mutational profile of top 20 mutated genes in candidate genes. Each column represents a patient, and each line represents a gene. The top bar graph shows the number of mutations detected in each sample and the mutation rate of significantly mutated genes is displayed on the right. Mutation types are indicated by the colors.

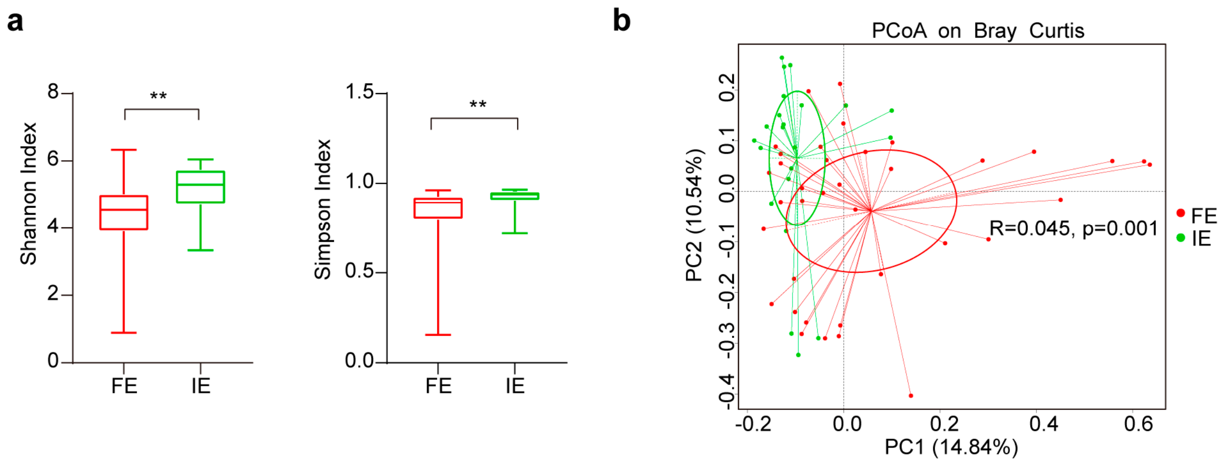

Figure S2. Bacterial community structure in FE (n = 37) and IE (n = 22) group. (**a**) Alpha diversity indices (Shannon index, Simpson's index) by Wilcoxon rank-sum test, *$p < 0.05$, **$p < 0.01$. (**b**) Principal coordinates analysis (PCoA) based on Bray–Curtis's dissimilarity. ADONIS analysis showed that the separation of bacterial communities was significant ($p < 0.05$). FE frequent exacerbator, IE infrequent exacerbator
